# Cortisol metabolism in pregnancies with small for gestational age neonates

**DOI:** 10.1038/s41598-019-54362-0

**Published:** 2019-11-29

**Authors:** Anna Siemiątkowska, Katarzyna Kosicka, Agata Szpera-Goździewicz, Mariola Krzyścin, Grzegorz H. Bręborowicz, Franciszek K. Główka

**Affiliations:** 1 Department of Physical Pharmacy and Pharmacokinetics, Poznan University of Medical Sciences, 6 Święcickiego Street, 60-781 Poznań, Poland; 2 Department of Perinatology and Gynecology, Poznan University of Medical Sciences, 33 Polna Street, 60-535 Poznań, Poland

**Keywords:** Pre-eclampsia, Endocrine reproductive disorders

## Abstract

Small for gestational age (SGA) newborns are often born from hypertensive pregnancies. This study aimed to compare the systemic metabolism of cortisol (F) in pregnancies with SGA and appropriate for gestational age (AGA) infants, considering both the normotensive (NT) and hypertensive patients. We hypothesized that the disturbances in systemic metabolism of F in pre-eclampsia (PE) might be attributed not to hypertension only, but to SGA. The study included 117 pregnants in the third trimester, divided into groups: NT pregnancy and SGA neonate (SGA-NT); NT pregnancy and AGA neonate (AGA-NT; controls), and respective groups with PE: SGA-PE and AGA-PE. We assessed the glucocorticoid balance with the function of enzymes involved in systemic metabolism of F: 11β-hydroxysteroid dehydrogenase type 1 and 2 (11β-HSD1 and 11β-HSD2), 5α- and 5β-reductase. The enzymes’ functions were estimated with the levels of F, cortisone (E), and their metabolites in plasma or urine, which we measured with HPLC-FLD and HPLC-MS/MS. The plasma F/E and urinary free F/E (UFF/UFE) ratios correlated significantly only in patients with the normal function of 5α- and 5β-reductase. The increased function of 11β-HSD2 was noted in all pre-eclamptic pregnancies. Increased function of 5α- and 5β-reductase was specific only for SGA-PE pregnancies, and the function of 5α-reductase was dependent on fetal sex. The SGA-NT pregnancies with male fetuses trended towards the higher function of renal 11β-HSD2 and 5β-reductase; SGA-NT pregnancies with female fetuses lacked any systemic glucocorticoid imbalance. In conclusion, systemic metabolism of F is the most intensive in pre-eclamptic pregnancies complicated by SGA with female fetuses. Our study supports the hypothesis about the different origins of PE and idiopathic intrauterine growth restriction and suggests the sex-specific mechanisms responsible for fetal growth restriction.

## Introduction

Newborns born with the birth weight below the 10^th^ percentile concerning their sex and gestational age (GA) at delivery are classified as small for gestational age (SGA). Low birth weight may result from the intrauterine growth restriction (IUGR) in which the fetus fails to achieve its full growth potential^1^. Both IUGR and SGA pregnancies correlate with the higher rates of neonatal morbidity and mortality, lower Apgar scores, placental abruption, pre-term deliveries, and stillbirth^2,3^. Moreover, lower birth weight increases the risk of cardiovascular, metabolic and mental disorders in adulthood^1,4^.

More than 60% of SGA pregnancies have an unexplained etiology and remain idiopathic^2^. Low birth weight may be a consequence of the constitutional factors such as lower maternal weight; SGA may also result from pathological conditions. Risk factors that promote lower birth weight could be of environmental, maternal, fetal, and placental origin^1,5^. Hypertensive pregnancy, especially pre-eclampsia (PE), is one of them. It attributes to 12–25% of all SGA/IUGR cases^6^.

Association between SGA/IUGR and PE and the disturbed activity and/or expression of placental 11β-hydroxysteroid dehydrogenase type 2 (11β-HSD2) has been established^7–12^. The enzyme protects the fetus from the high concentration of maternal cortisol (F) due to inactivation of F into cortisone (E)^13^. We have previously reported^14,15^ the increased apparent activity of renal 11β-HSD2 in women with gestational hypertension and PE – conditions which are often complicated by the SGA neonates^16^. Therefore, the question emerged whether the observed abnormalities occur only in the hypertensive pregnancy and if they also are present in IUGR.

A part of the answer came in 2018 with the study of *Vasku et al*.^17^. They analyzed serum F/E ratios in pregnant women and concluded about higher systemic 11β-HSD2 activity in PE without IUGR but not in IUGR without PE. However, the ratio of urinary free F (UFF) and free E (UFE) indicates the most reliably the 11β-HSD2 function^18^. Furthermore, the 11β-HSD2 is the only one of the four major enzymes involved in the metabolism of F; to evaluate the glucocorticoid balance properly, one should also consider the function of 11β-hydroxysteroid dehydrogenase type 1 (11β-HSD1), 5α- and 5β-reductase^19–22^.

The scarce data concern the systemic function of these enzymes and the UFF/UFE in relation to SGA pregnancies. Disturbed expression or activity of 11β-HSD1 was observed in the placenta, fetal membranes, or decidua of PE/IUGR pregnancies^23–25^. We previously indicated the imbalance in the function of 5α/β-reductase in PE^15^; no published work discussed the issue in gestations complicated by SGA.

This study aimed to estimate and compare the apparent activities of 11β-HSD1, 11β-HSD2, 5α- and 5β-reductase in SGA and appropriate for gestational age (AGA) pregnancies. We hypothesized that the disturbances in systemic metabolism of F in PE might be attributed not to hypertension only, but to SGA, which often accompanies PE. Glucocorticoid balance was analyzed in both normotensives and pre-eclamptic women with an emphasis on any abnormalities specific to SGA condition. We assessed the function of the enzymes by the levels of plasma and urinary F, E, and their tetrahydro- and allo-tetrahydrometabolites (THF and allo-THF for F; THE and allo-THE for E) in urine.

## Materials and Methods

### Study population

The study was performed in accordance with the Declaration of Helsinki. The Ethical Committee at Poznan University of Medical Sciences approved the protocol (documents no. 954/11 and 1129/16). Each participant gave written informed consent before including in the project. We enrolled 117 pregnant women hospitalized in Gynecological and Obstetrics University Hospital of Poznan University of Medical Sciences between 2013 and 2016. All subjects were of Polish Caucasian origin and had a singleton pregnancy with GA ranging from 27 to 41.

The study population comprised women who gave birth to a child classified as SGA, based on the newborn’s birth weight (lower than the 10^th^ percentile as defined by the American College of Obstetricians and Gynecologists^26^) according to the regional data^27^. The fact of SGA was suspected during pregnancy, considering the estimated fetal weight (pregnancies with IUGR^26^), but was finally confirmed after delivery. For comparative purposes, two types of patients were included: normotensive and pre-eclamptic ones. They constituted two different groups (SGA-NT and SGA-PE, respectively) and were analyzed separately.

The results of women from SGA groups were compared to AGA (newborn’s birth weight between 10^th^ and 90^th^ percentile for appropriate GA and sex)^27^. The AGA groups (AGA-NT and AGA-PE) were matched to proper SGA groups (SGA-NT and SGA-PE, respectively) regarding GA at sample collection, maternal pre-pregnancy BMI, maternal age, and parity. The exclusion criteria for all study groups comprised: stillbirth and congenital fetal defects, chromosomal anomalies, chronic infectious diseases, mental and chronic liver disorders, and metabolic and endocrine diseases (except for hypothyroidism and gestational diabetes). Patients who declared consuming a significant amount of licorice products or grapefruit juice, being active smokers, or with a history of alcohol or drug abuse were also not eligible. Thus, our SGA-NT group comprised women with idiopathic SGA (IUGR) babies while AGA-NT group consisted of healthy controls.

Despite the complexity of PE, we defined the condition as hypertension (blood pressure ≥ 140/90 mmHg from 2 measurements, at least 4 h apart) and new-onset of proteinuria ≥ 300 mg/24 h^28^. This approach is the most common in scientific research, and we maintained the criteria for better comparability with previously published results. BMI before pregnancy was calculated from self-reported weight and height, while prematurity was defined as giving birth before the 37th week of gestation. Women diagnosed with PE were treated with antihypertensive drugs, mostly with methyldopa (>94% of pre-eclamptic patients) - either as an independent therapy or in combination with other medications (nitrendipine, metoprolol, verapamil, magnesium sulfate) while those with hypothyroidism took levothyroxine.

Table 1 shows the general characteristics of patients. There were no significant differences between 4 study groups regarding maternal age, GA at sampling, nulliparity, infant’s sex, or frequency of suffering from gestational diabetes and hypothyroidism. Two patients from the AGA-PE group (10.5%) had asthma but did not take steroids during gestation. Six women from each of the pre-eclamptic groups (SGA-PE and AGA-PE) were previously diagnosed with chronic hypertension; the others suffered from pregnancy-induced hypertension. Prematurity was recognized in 73.7% of cases from SGA-PE group, 36.8% of AGA-PE, 28.6% of SGA-NT and only 6.4% of AGA-NT. Among PE patients there was a marked disparity in proteinuria of 2.11 (0.52–3.26) vs. 0.46 (0.35–1.05) g/24 h (medians (interquartile ranges)) for SGA-PE and AGA-PE group, respectively.

**Table 1:** Characteristics of the study population.

	normotensive women		hypertensive women		P-value*
	AGA-NT	SGA-NT	AGA-PE	SGA-PE	
	(n = 50)	(n = 29)	(n = 19)	(n = 19)	
Age^1^ [y]	30.3 ± 4.5	28.5 ± 5.1	32.2 ± 4.1	31.5 ± 6.3	NS
Nulliparity^2^	22 (44.0%)	16 (55.2%)	13 (68.4%)	12 (63.2%)	NS
BMI before pregnancy^3^ [kg/m^2^]	21.3 (19.6–23.8)^c^	20.0 (19.1–21.8)^c,d^	24.5 (21.4–27.6)^a,b^	23.2 (21.1–25.7)^b^	*P* = 0.0009
**Concomitant diseases**^2^					
• hypothyroidism • gestational diabetes	6 (12.0%) 4 (8.0%)	3 (10.3%) 1 (3.4%)	4 (21.0%) 4 (21.0%)	2 (10.5%) 3 (15.8%)	NS NS
GA at sample collection^3^ [wks]	36 (32–38)	34 (31–37)	35 (30–38)	33 (30–35)	NS
GA at delivery^3^ [wks]	39 (38–40)^d^	38 (36–39)^d^	37 (33–40)^d^	34 (33–37)^a,b,c^	*P* < 0.0001
Infant birth weight^3^ [g]	3330 (2950–3610)^b,d^	2455 (1675–2620)^a^	2910 (2030–3250)^d^	1640 (1390–1850)^a,c^	*P* < 0.0001
Female fetuses^2^	18 (38.3%)	15 (53.6%)	9 (47.4%)	8 (42.1%)	NS

Results are presented as: ^1^mean ± SD, ^2^number of patients (%), ^3^median (interquartile range); *comparison was performed with ANOVA, Kruskal-Wallis test or χ^2^-test, as appropriate.

^a^*P* < 0.05 compared with a normotensive pregnancy with appropriate for gestational age baby (AGA-NT group = controls).

^b^*P* < 0.05 compared with a normotensive pregnancy complicated by small for gestational age baby (SGA-NT group).

^c^*P* < 0.05 compared with a pre-eclamptic pregnancy with appropriate for gestational age baby (AGA-PE group).

^d^*P* < 0.05 compared with a pre-eclamptic pregnancy complicated by small for gestational age baby (SGA-PE group).

### Samples and methods

Each patient provided one fasting blood sample and a 24-hour urine collection. The blood was collected in the morning (at 7.00–8.00 a.m.) on the day when the urine collection was completed. The blood sample was centrifuged at 1740 × *g* for 10 min, and plasma was collected. Both plasma and urine were stored at −25 °C until analyzed. The HPLC-FLD method was used to determine the total concentrations of F and E in plasma, as described in detail elsewhere^14^. Urinary UFF and UFE, as well as total amounts of F, E, THF, allo-THF, THE, and allo-THE, were measured with HPLC-MS/MS method^29^. For total steroids, assays were preceded by enzymatic hydrolysis (37 °C, 20 h) with β-glucuronidase from *Helix pomatia*. When the determined steroids were below the lower limit of quantitation or the limit of detection, we applied the previously described procedure^15^. Based on the available literature^18,20–22,30,31^, the apparent activity of enzymes involved in the metabolism of F was assessed by: UFF/UFE for 11β-HSD2 function; (THF + allo-THF)/(THE + allo-THE) noted as THFs/THEs for global 11β-HSD activity; allo-THF/F for 5α-reductase; and THF/F for 5β-reductase. In calculations regarding 5α- and 5β-reductase function, we used only the total glucocorticoids. This decision was made because of our previous observations^15^ concerning the disturbed conjugation degree of certain steroids with glucuronic and sulphuric acid in pre-eclamptic pregnancies.

Additionally, the F metabolic clearance was calculated by (THFs + THEs)/UFF ratio^18^; the net glucocorticoid balance by plasma F/E ratio^32^; the daily secretion of F by the sum of THFs, THEs, total F and total E in urine^31^.

### Statistics

The statistical analysis was carried out using Statistica 13 software (Statsoft Inc., Tulsa, OK, USA). The continuous variables were expressed, depending on the results of the Shapiro-Wilk test, as a mean ± SD or a median score (interquartile range), while the categorical data as a number of subjects (%). The differences between groups were checked with ANOVA, Kruskal-Wallis/Mann-Whitney U test, or χ^2^ test for normally distributed, non-parametric, and categorical data, respectively. The simple correlations were assessed with the Spearman test. In each analysis, a *P*-value < 0.05 was considered significant.

The use of the Kruskal-Wallis test enabled to identify both the influence of PE/SGA on F metabolism as well as the interaction between PE and SGA. However, patients with PE presented higher pre-pregnancy BMI compared with normotensive individuals. Therefore, the forward multiple regression analyses were performed (selection F = 1, elimination F = 0) to particularize the steroid results from the Kruskal-Wallis test.

## Results

### Glucocorticoid balance in pre-eclamptic pregnancies

**Figure 1:**
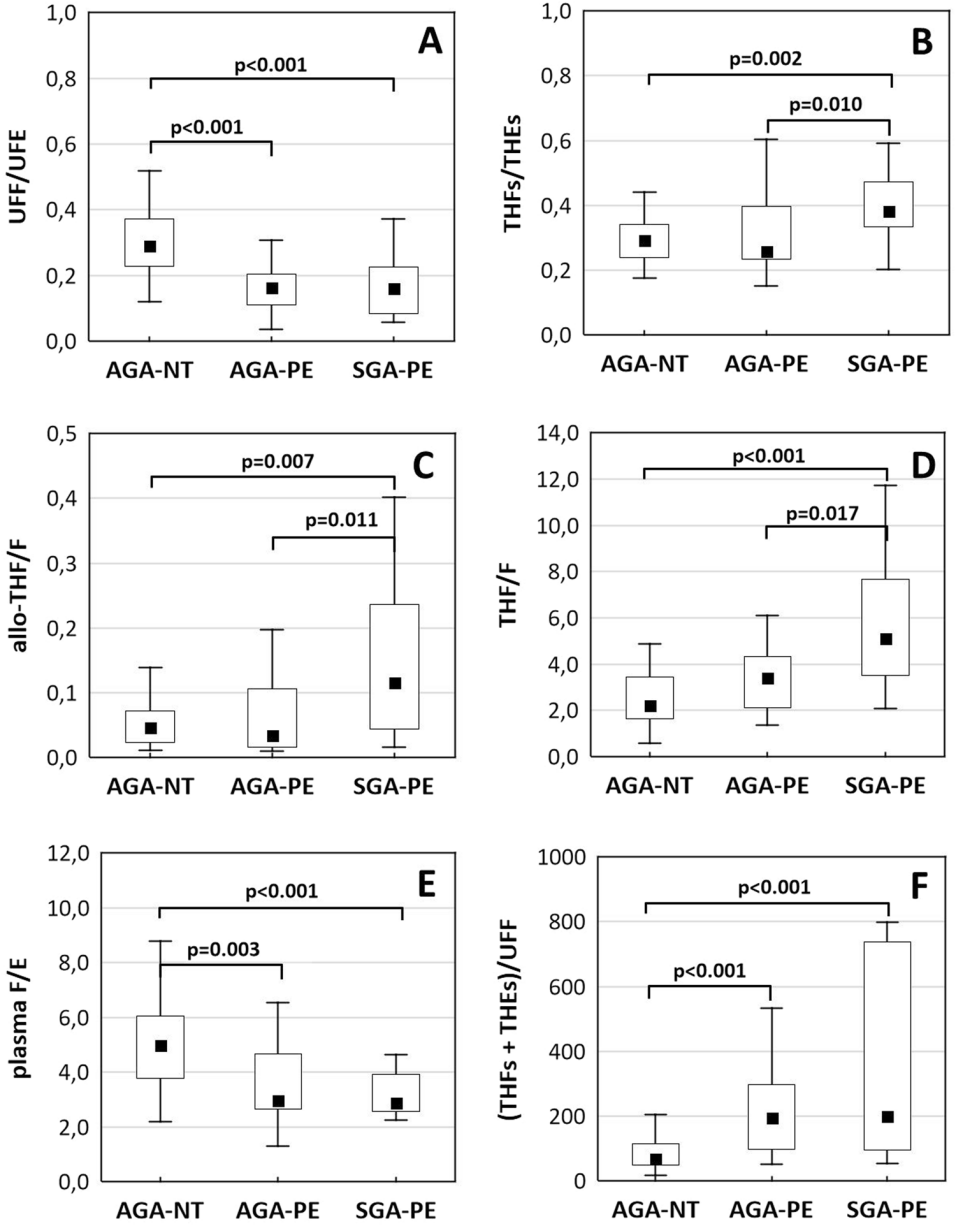
Glucocorticoid balance in pregnant women: normotensive with appropriate for gestational age newborn (AGA-NT), as well as pre-eclamptic with appropriate or small for gestational age newborns (AGA-PE and SGA-PE, respectively). The function of renal 11β-HSD2 (reflected by the ratio of urinary UFF/UFE – **A**), systemic 11β-HSD (THFs/THEs – **B)**, hepatic 5α- and 5β-reductase (allo-THF/F - **C**; THF/F - **D**, respectively), overall balance between F and E (plasma F/E - **E**) as well as the F clearance [(THFs + THEs)/UFF - **F**]. Boxplots present: median (middle point), interquartile range (box), and range. Outliers were excluded according to Tuckey’s method. The detailed P-values for between-group differences are presented in the figure.

The analysis of glucocorticoid balance concerning fetal sex did not show significant differences in the three groups (AGA-NT, AGA-PE, and SGA-PE; supplementary data - Figs. S1–S6). Therefore, for pre-eclamptic pregnancies, we made all further comparisons for combined groups (comprising both female and male fetuses). Figure 1 presents the calculated parameters reflecting the function of 11β-HSD2, global 11β-HSD, 5α- and 5β-reductase, as well as the overall glucocorticoid balance in the body (AGA-NT vs. AGA-PE vs. SGA-PE). The detailed results (levels of plasma and urinary steroids and calculated ratios) are shown in supplementary Table S1.

The total secretion of glucocorticoids [(THFs + THEs + F + E)/UCr ratio] was similar in all groups (Table S1). Both pre-eclamptic groups (AGA-PE and SGA-PE) presented higher apparent activity of 11β-HSD2 (lower UFF/UFE; Fig. 1A), higher metabolic clearance of F [higher (THFs + THEs)/UFF; Fig. 1F] and lower plasma F/E (Fig. 1E) compared with healthy controls (AGA-NT group). Pre-eclamptic pregnancies complicated by the SGA baby (SGA-PE group) differed significantly from those with the AGA neonates (AGA-PE) and showed: the increased function of 5α-reductase (higher allo-THF/F; Fig. 1C), 5β-reductase (higher THF/F; Fig. 1D) and changed global 11β-HSD (higher THFs/THEs; Fig. 1B).

### Glucocorticoid balance in SGA pregnancies

**Figure 2:**
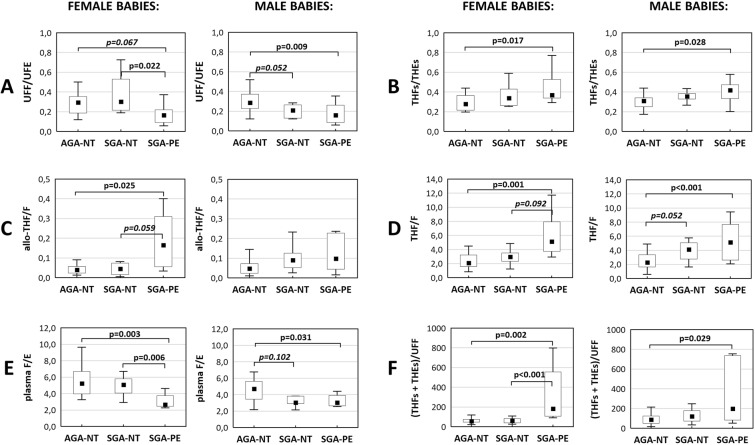
Glucocorticoid balance in pregnant women: normotensive with appropriate or small for gestational age newborn (AGA-NT and SGA-NT, respectively), as well as pre-eclamptic with small for gestational age newborns (SGA-PE). The results are presented separately for pregnancies with female or male fetuses. The function of renal 11β-HSD2 (UFF/UFE - **A**), systemic 11β-HSD (THFs/THEs - **B**), hepatic 5α- and 5β-reductase (allo-THF/F - **C**; THF/F - **D**, respectively), overall balance between F and E (plasma F/E - **E**) as well as the F clearance [(THFs + THEs)/UFF - **F**]. Boxplots present: median (middle point), interquartile range (box), and range. Outliers were excluded according to Tuckey’s method. The detailed P-values for between-group differences are presented in the figure.

The analysis of glucocorticoid balance concerning fetal sex showed significant differences only in the SGA-NT group (supplementary data – Figs. S1–S6). Therefore, we made all further comparisons separately for pregnancies with female or male fetuses. Results of Kruskal-Wallis analyses (AGA-NT vs. SGA-NT vs. SGA-PE) concerning fetal sex are presented in Fig. 2. Detailed results are attached in supplementary materials (Tables S2 and S3).

The total secretion of glucocorticoids was similar in all three groups (Tables S2 and S3). The trend towards the higher function of 11β-HSD2, which is characteristic for pre-eclamptic pregnancies, can also be observed in SGA-NT, but only with male fetuses (*P* = *0*.*052*; Fig. 2A). A similar situation can be observed with plasma F/E (*P* = *0*.*102*; Fig. 2E). The female SGA-NT group presents values of urinary UFF/UFE (Fig. 2A) and plasma F/E (Fig. 2E) comparable to AGA-NT and significantly higher than female SGA-PE. Metabolic clearance of F is significantly higher only in pre-eclamptic pregnancies (SGA-PE), SGA-NT pregnancies are comparable to AGA-NT (both with female and male fetuses; Fig. 2F). Increased functions of 5α- and 5β-reductases, observed in SGA-PE pregnancies, are determined by fetal sex. Increased function of 5α-reductase (Fig. 2C) is specific only for pre-eclamptic pregnancies (SGA-PE) with female fetuses. On the contrary, the function of 5β-reductase (Fig. 2D) is increased in both female and male SGA-PE pregnancies. SGA-NT group with only male fetuses showed increased function of 5β-reductase (*P* = *0*.*052*). Tables S4–S6 show the results from multiple regression analyses, which confirm and complete the Kruskal-Wallis test.

**Figure 3:**
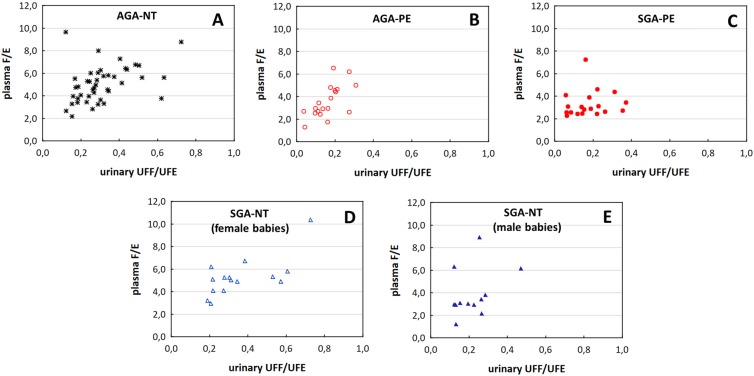
The correlations between plasma F/E ratio and urinary UFF/UFE ratio in AGA-NT (asterisks; **A**), AGA-PE (empty circles; **B**), SGA-PE (full circles; **C**), SGA-NT group with FEMALE fetuses (empty triangles; **D**), and SGA-NT group with MALE fetuses (full triangles; **E**). The Spearman test showed significant correlations between calculated parameters in AGA-NT (R = 0.479; *P* = 0.001), AGA-PE (R = 0.651; *P* = 0.002), and in SGA-NT with FEMALE fetuses (R = 0.593; *P* = *0*.*020*). No correlation was observed either in SGA-PE group or in SGA-NT with MALE fetuses.

Figure 3 presents the results of the Spearman test; plasma F/E correlates with urinary UFF/UFE ratio in the AGA-NT group (R = 0.479), AGA-PE group (R = 0.651) and SGA-NT subgroup with female babies (R = 0.593), but neither in SGA-PE group nor in SGA-NT subgroup with male babies.

## Discussion

Our previous studies revealed the increased function of systemic 11β-HSD2^14,15^, 5α-reductase, and 5β-reductase^15^ in PE compared with a healthy pregnancy. Here, we present novel findings on the functions of 5α- and 5β-reductase. The increase in functions of 5α- and 5β-reductase are characteristic for pre-eclamptic gestations only with SGA baby and additionally are associated with the fetal sex. The increase in 5α-reductase function relates to SGA-PE pregnancies only with female fetuses, while the function of 5β-reductase increases in all SGA-PE pregnancies. The apparent activity of 11β-HSD2 increases in all pregnancies with PE, irrespectively either of the growth of the fetus (both SGA-PE and AGA-PE) or the fetal sex.

The function of 11β-HSD2 in PE increases regardless of the SGA condition: the UFF/UFE ratio was significantly lower in both pre-eclamptic groups (SGA-PE and AGA-PE) in comparison with healthy controls (Fig. 1A). Our results are consistent with those presented by *Vasku et al*.^17^ and *Jayasuriya et al*.^33^, and detail the glucocorticoids’ profile in pre-eclamptic pregnancy. The advantage of our study is that we included the two cohorts of pre-eclamptic women: those who gave birth to SGA (SGA-PE), and AGA neonates (AGA-PE).

The urinary THFs/THEs ratio was higher in the SGA-PE group than in controls and AGA-PE (Fig. 1B). Provided the normal activities of 11β-HSD2 and 5α/β-reductase, the F and E metabolites ratio usually predicts the function of 11β-HSD1^20,22,31^. In our study, the conclusions about 11β-HSD1 cannot be derived from the THFs/THEs - the THFs/THEs value in SGA-PE is impacted by the higher systemic activities of 11β-HSD2 (Fig. 1A), 5α-reductase (Fig. 1C) and 5β-reductase (Fig. 1D). *Finken et al*. reported similar situation when comparing the F metabolism in women and men^22^. The authors attributed the higher THFs/THE ratio rather to the different activity of 5β-reductase than to the abnormal activity of 11β-HSD1.

In the non-pregnant population, approx. 50% of F, secreted by the adrenal cortex, appear in the urine as A-ring metabolites^19^. A sum of THFs, THEs and the total amount of F and E in urine emerged as a reliable parameter to assess the daily production of F^31^. This index was similar in all study groups (Tables S1–S3). It suggests that despite the increased systemic metabolism of F in pre-eclamptic gestation (reflected in the higher clearance of F; Fig. 1F), the overall secretion of F remains unchanged.

The SGA-PE group, but not AGA-PE, manifested lower plasma F concentration (Table S1). Such results may indicate the blunted HPA axis in SGA-PE. The limitation of this assumption is a single measurement of total plasma F (morning sample); therefore, it needs verification in the future. Some authors noted the decreased plasma F in PE^33–35^, but one has never assessed this phenomenon in relation to the coexisting PE and SGA. *Vasku et al*. demonstrated similar levels of serum F in PE without IUGR and healthy controls^17^. This finding is in agreement with our observations for the AGA-PE group.

Scarce literature data concern the systemic F metabolism in women with idiopathic IUGR. Idiopathic IUGR was characterized by normal values of serum F/E ratio^17,33^, plasma E/F ratio^10^, or serum levels of A-ring metabolites of F and E^17^. It complies with our findings from plasma and urine for the SGA-NT group with female fetuses. None of the cited authors^10,17,33^ analyzed glucocorticoid balance in idiopathic IUGR pregnancy concerning fetal sex. Our results indicate that it may be fetal-sex-specific: we revealed the trend towards increased function of renal 11β-HSD2 (Fig. 2A) and 5β-reductase (Fig. 2D) in SGA-NT subgroup with males (Tables S2–S3). No such trend occurred in SGA-NT subgroup with female fetuses. Differences in maternal glucocorticoid metabolism, which are fetal-sex-dependent, add to the topic on sex-specific differences in early-life programming of HPA axis^36^.

IUGR and PE might have similar etiology as they both result from placental insufficiency. On the other hand, PE and idiopathic IUGR have different risk factors^37^, and associate with various abnormalities^38–40^. Our study supports the hypothesis on the different origins of IUGR and PE and suggests the various molecular mechanisms responsible for growth restriction depending on the fetal sex. The data on sex-specific differences in function or expression of 11β-HSD1 and 11β-HSD2 are inconclusive. Some authors reported a lack of sex-specific differences in 11β-HSD1/11β-HSD2 expression or activity in placentas in the SGA or control group^10,24^. Others showed the lower activity of 11β-HSD2 in placentas from SGA pregnancies with female fetuses when compared to those with male fetuses^41^. Also, antenatal betamethasone administration, which usually reduces newborn’s birth weight, is linked with the decreased protein expression and activity of placental 11β-HSD2 only in pregnancies with female fetuses^42^. We observed that UFF/UFE ratio in normotensive mothers with female SGA fetuses is higher than with male SGA fetuses (Fig. S1). This observation supports the findings of *Mericq et al*.^41^ and indicates that the trend towards the lower function of 11β-HSD2 in SGA-NT pregnancies with females concerns both placental and renal 11β-HSD2. A sex-specific difference in the activity of cortisol-metabolizing enzymes was also observed in the pre-eclamptic SGA group. The increased function of 5α-reductase was noted only in the female subgroup (Fig. 2C). According to our best knowledge, there is no report concerning systemic function of 5α-reductase in pregnancies with male vs. female fetuses. Higher activity of 5α-reductase was however noted in placental tissue from male pregnancies^43^.

Despite plasma or serum F/E ratio is a non-specific parameter that reflects the overall glucocorticoid balance in the body, it expresses more accurately the activity of 11β-HSD2 than 11β-HSD1^44^. It is used to calculate the 11β-HSD2 function in non-pregnant^32^ and pregnant population^17^. We assessed the correlation between plasma F/E ratio and urinary UFF/UFE – the best surrogate marker for the activity of renal 11β-HSD2^18^. The significant correlation occurred in AGA-NT group, AGA-PE group and SGA-NT subgroup with female fetuses. These groups were found to have normal function of 5α/β-reductases (Tables S1–S3). A correlation was found neither in SGA-PE group nor in SGA-NT subgroup with male fetuses (Fig. 3) – both characterized by the increased A-ring production (as described above). Pregnant women may exhibit many disturbances in glucocorticoid balance, both physiological (hypercortisolism or the additional source of 11β-HSD2 – the placenta^13^) and pathological (discussed above). We state that the clear association between plasma F/E and the function of renal 11β-HSD2 exists only in case of the unaffected function of other enzymes involved in the metabolism of F.

The limitation of our study is that the study groups differed in terms of pre-pregnancy BMI. The AGA groups matched to respective SGA groups for BMI (AGA-NT to SGA-NT and AGA-PE to SGA-PE), but women with PE presented higher BMI than normotensives. A similar situation was reported by other authors who studied patients with PE, isolated IUGR, and healthy controls^40,45^. This finding can be explained by the risk factors of PE (higher maternal BMI^37,46,47^) and SGA infants (lower BMI^48^).

## Conclusions

Our results indicate that PE in pregnancy is associated with the intensified systemic metabolism of F, further increased in co-existing SGA, especially with female fetuses. We suggest it may result from a compensatory mechanism in response to an excess of proapoptotic F, which reaches the fetus. Such a phenomenon is absent in idiopathic SGA pregnancies with female fetuses, which supports the hypothesis about the different origins of PE and normotensive IUGR. The observed disparities also confirm the existence of various types of PE.

Our main findings include: **(i)** Compared with healthy controls, the increased function of 5α- and 5β-reductase occurs only in pre-eclamptic SGA pregnancies, while the increased systemic apparent activity of 11β-HSD2 - in all pre-eclamptic pregnancies; the activity of 5α-reductase in SGA-PE is sex-dependent with a much higher function in pregnancies with female than male fetuses; **(ii)** Idiopathic (normotensive) SGA pregnancies exhibit sex-specific differences in the function of enzymes involved in the metabolism of F: compared with healthy controls, pregnancies with males trend towards higher function of renal 11β-HSD2 and 5β-reductase; normotensive SGA pregnancies with female fetuses lack any systemic glucocorticoid imbalance; **(iii)** In pregnant women, plasma F/E ratio correlates significantly with UFF/UFE ratio only in cases with the proper function of 5α- and 5β-reductases - when considered as an index of the apparent activity of 11β-HSD2, plasma F/E should be used with a great caution; **(iv)** Daily production of F changes neither in pre-eclamptic nor in idiopathic SGA pregnancy.

## Supplementary information

Supplementary Materials
