# Supplementary material for: Cortisol metabolism in pregnancies with small for gestational age neonates
Source: Sci Rep. 2019 Nov 29;9:17890. doi: 10.1038/s41598-019-54362-0 (PMC6884581; doi:10.1038/s41598-019-54362-0)

Supplementary materials

Cortisol metabolism in pregnancies with small for gestational age neonates

Anna SIEMIĄTKOWSKA[a], Katarzyna KOSICKA[a*], Agata SZPERA-GOŹDZIEWICZ[b], Mariola KRZYŚCIN[b], Grzegorz H. BRĘBOROWICZ[b], Franciszek K. GŁÓWKA[a]

[a] Department of Physical Pharmacy and Pharmacokinetics, Poznan University of Medical Sciences, 6 Święcickiego Street, 60-781 Poznań, Poland

[b] Department of Perinatology and Gynecology, Poznan University of Medical Sciences, 33 Polna Street, 60-535 Poznań, Poland

AGA – appropriate for gestational age; allo-THE – 5α-tetrahydrocortisone, allo-tetrahydrocortisone; alloTHF – 5α-tetrahydrocortisol, allo-tetrahydrocortisol; BMI – body mass index; E – cortisone; F – cortisol; GA – gestational age; NS – not significant; NT – normotensive; PE – pre-eclampsia; SGA – small for gestational age; THE – 5β-tetrahydrocortisone, tetrahydrocortisone; THEs – sum of total THE and allo-THE in urine; THF – 5β-tetrahydrocortisol, tetrahydrocortisol; THFs – sum of total THF and allo-THF in urine; UCr – urinary creatinine; UFE – urinary free cortisone; UFF – urinary free cortisol.

Table S1. Glucocorticoid balance in pregnant women: normotensive with appropriate for gestational age newborn (AGA-NT), as well as pre-eclamptic with appropriate or small for gestational age newborns (AGA-PE and SGA-PE, respectively). The parameters calculated from the levels of glucocorticoid in plasma and urine are marked in bold.

	AGA-NT	AGA-PE	SGA-PE	*P*-value*
	(n = 43)	(n = 19)	(n = 19)	
plasma F [nmol/L][1]	789.7 (690.9-957.0)[c]	722.3 (444.2-834.4)	645.4 (518.1-852.8)[a]	*P*=0.0118
plasma E [nmol/L][1]	172.1 (139.1-204.7)	171.0 (154.4-252.2)	198.8 (117.4-251.4)	NS
**plasma F/E**	**4.98 (3.77-6.04)[b,c]**	**2.96 (2.65-4.66)[a]**	**2.89 (2.56-3.91)[a]**	***P*<0.0001**
	(n = 50)	(n = 19)	(n = 19)	
UFF/UCr [µg/mmol][2]	5.97 (4.16-7.47)[b,c]	2.70 (1.89-4.34)[a]	3.02 (0.81-4.73)[a]	*P*<0.0001
UFE/UCr [µg/mmol][2]	19.90 (13.19-25.59)	18.19 (13.22-26.39)	13.23 (6.82-18.81)	NS
**UFF/UFE**	**0.290 (0.229-0.373)[b,c]**	**0.162 (0.111-0.204)[a]**	**0.161 (0.085-0.227)[a]**	***P*<0.0001**
F/UCr [µg/mmol][3]	36.70 (30.70-48.23)[c]	31.60 (27.23-39.36)	32.16 (10.03-36.30)[a]	*P*=0.0197
E/UCr [µg/mmol][3]	36.38 (29.19-40.47)[c]	29.90 (20.26-40.83)	27.58 (12.14-32.64)[a]	*P*=0.0036
allo-THF/UCr [µg/mmol][3]	1.86 (0.96-2.78)	0.85 (0.70-3.40)	1.48 (0.95-3.96)	NS
THF/UCr [µg/mmol][3]	84.1 (67.1-122.5)	103.7 (51.5-153.3)	113.1 (66.67-159.5)	NS
allo-THE/UCr [µg/mmol][3]	2.90 (1.90-5.10)	2.52 (1.62-3.78)	2.37 (0.96-4.53)	NS
THE/UCr [µg/mmol][3]	295.9 (223.7-378.4)	338.4 (194.5-599.0)	238.8 (147.9-553.4)	NS
**THFs/THEs**	**0.293 (0.239-0.341)[c]**	**0.259 (0.234-0.397)[c]**	**0.382 (0.334-0.473)[a,b]**	***P*=0.0015**
**allo-THF/F**	**0.0467 (0.0234-0.0717)[c]**	**0.0346 (0.0160-0.1053)[c]**	**0.1155 (0.0442-0.2362)[a,b]**	***P*=0.0039**
**THF/F**	**2.21 (1.64-3.45)[c]**	**3.40 (2.13-4.31)[c]**	**5.13 (3.51-7.69)[a,b]**	***P*<0.0001**
**(THFs + THEs)/UFF**	**67.8 (48.8-114.4)[b,c]**	**194.2 (98.0-297.4)[a]**	**199.2 (94.5-737.4)[a]**	***P*<0.0001**
**(THFs+THEs+F+E)/UCr**	**468.0 (373.7-578.0)**	**595.5 (278.8-815.4)**	**424.9 (292.0-783.9)**	**NS**
urine volume [ml/24 h]	1555 (1150-1940)	1710 (1120-2780)	2050 (1200-3020)	NS
UCr [mmol/24 h]	8.13 (6.80-10.05)	10.32 (8.19-11.54)	9.01 (7.57-11.08)	NS

Results are presented as medians (interquartile ranges); [1] total plasma steroids, [2] free urinary steroids, [3] total urinary steroids. Free and total urinary steroids are corrected each time for UCr; *comparison was performed with the Kruskal-Wallis test with the post-hoc Dunn's test;

[a] *P*<0.05 compared with a normotensive pregnancy with appropriate for gestational age baby (AGA-NT group = **healthy controls**)

[b] *P*<0.05 compared with a pre-eclamptic pregnancy with appropriate for gestational age baby (AGA-PE group)

[c] *P*<0.05 compared with a pre-eclamptic pregnancy complicated by small for gestational age baby (SGA-PE group).

Table S2. Glucocorticoid balance in pregnant women with FEMALE fetuses: normotensive with appropriate or small for gestational age newborn (AGA-NT and SGA-NT, respectively), as well as pre-eclamptic with small for gestational age newborns (SGA-PE). The parameters calculated from the levels of glucocorticoid in plasma and urine are marked in bold.

	AGA-NT	SGA-NT	SGA-PE	*P*-value*
	(n = 14)	(n = 15)	(n = 8)	
plasma F [nmol/L][1]	779.1 (717.0-892.5)[c]	795.7 (562.9-986.6)	635.6 (333.1-685.6)[a]	*P*=0.0339
plasma E [nmol/L][1]	151.6 (126.1-201.3)	151.7 (129.3-196.5)	178.6 (87.8-246.9)	NS
**plasma F/E**	**5.25 (3.98-6.70)[c]**	**5.08 (4.08-5.79)[c]**	**2.68 (2.45-3.77)[a,b]**	***P*=0.0018**
	(n = 18)	(n = 15)	(n = 8)	
UFF/UCr [μg/mmol][2]	6.66 (4.79-8.06)[c]	7.27 (4.95-10.04)[c]	2.61 (0.47-4.40)[a,b]	*P*=0.0011
UFE/UCr [μg/mmol][2]	21.94 (16.54-28.26)[c]	18.81 (16.96-27.10)	12.37 (4.48-18.33)[a]	*P*=0.0210
**UFF/UFE**	**0.296 (0.185-0.354)**	**0.303 (0.216-0.530)[c]**	**0.165 (0.091-0.220)[b]**	***P*=0.0223**
F/UCr [μg/mmol][3]	34.30 (31.19-49.89)	32.69 (31.37-45.45)	25.55 (6.07-32.74)	NS
E/UCr [μg/mmol][3]	36.78 (31.05-43.82)[c]	31.25 (25.10-43.50)	23.63 (7.64-28.65)[a]	*P*=0.0055
allo-THF/UCr [μg/mmol][3]	1.49 (0.77-2.78)	1.51 (0.54-2.12)	1.87 (1.07-3.63)	NS
THF/UCr [μg/mmol][3]	75.1 (62.6-122.5)	95.0 (74.9-140.8)	113.1 (45.2-137.2)	NS
allo-THE/UCr [μg/mmol][3]	2.94 (1.67-6.74)	2.50 (1.97-4.17)	2.17 (0.71-6.52)	NS
THE/UCr [μg/mmol][3]	275.4 (223.7-413.2)	251.8 (209.0-466.5)	240.2 (140.4-331.3)	NS
**THFs/THEs**	**0.278 (0.215-0.364)[c]**	**0.339 (0.263-0.430)**	**0.369 (0.338-0.528)[a]**	***P*=0.0152**
**allo-THF/F**	**0.0407 (0.0231-0.0570)[c]**	**0.0450 (0.0155-0.0744)**	**0.1650 (0.0552-0.3103)[a]**	***P*=0.0231**
**THF/F**	**2.09 (1.61-3.22)[c]**	**2.97 (2.31-3.50)**	**5.13 (3.73-7.95)[a]**	***P*=0.0016**
**(THFs + THEs)/UFF**	**58.4 (46.6-76.8)[c]**	**63.2 (38.7-83.9)[c]**	**183.8 (106.5-556.6)[a,b]**	***P*=0.0004**
**(THFs+THEs+F+E)/UCr**	**465.1 (364.9-582.4)**	**399.1 (355.3-694.0)**	**424.7 (201.9-541.2)**	**NS**
urine volume [ml/24 h]	1705 (1190-1960)	2020 (1280-2430)	1800 (1335-2785)	NS
UCr [mmol/24 h]	7.44 (5.86-9.61)[c]	8.29 (7.40-9.22)	9.55 (8.82-13.37)[a]	*P*=0.0447

Results are presented as medians (interquartile ranges); [1] total plasma steroids, [2] free urinary steroids, [3] total urinary steroids. Free and total urinary steroids are corrected each time for UCr; *comparison was performed with the Kruskal-Wallis test with the post-hoc Dunn's test;

[a] $P<0.05$ compared with a normotensive pregnancy with appropriate for gestational age baby (AGA-NT group = **healthy controls**)

[b] $P<0.05$ compared with a normotensive pregnancy complicated by small for gestational age baby (SGA-NT group)

[c] $P<0.05$ compared with a pre-eclamptic pregnancy complicated by small for gestational age baby (SGA-PE group).

Table S3. Glucocorticoid balance in pregnant women with MALE fetuses: normotensive with appropriate or small for gestational age newborn (AGA-NT and SGA-NT, respectively), as well as pre-eclamptic with small for gestational age newborns (SGA-PE). The parameters calculated from the levels of glucocorticoid in plasma and urine are marked in bold**.**

	AGA-NT	SGA-NT	SGA-PE	*P*-value*
	(n = 26)	(n = 13)	(n = 11)	
plasma F [nmol/L][1]	791.0 (669.9-957.0)	699.4 (638.1-901.6)	645.4 (518.1-896.3)	NS
plasma E [nmol/L][1]	180.7 (146.3-213.6)	206.0 (158.0-263.6)	203.7 (117.4-251.4)	NS
**plasma F/E**	**4.73 (3.45-5.62)[c]**	**3.03 (2.92-3.81)**	**3.05 (2.65-3.91)[a]**	***P*=0.0142**
	(n = 29)	(n = 12)	(n = 11)	
UFF/UCr [µg/mmol][2]	5.60 (3.38-7.37)[c]	3.66 (2.72-5.53)	3.02 (1.40-4.73)[a]	*P*=0.0232
UFE/UCr [µg/mmol][2]	19.30 (12.07-24.52)	19.32 (10.45-28.20)	13.68 (8.69-23.87)	NS
**UFF/UFE**	**0.288 (0.229-0.373)[c]**	**0.211 (0.131-0.265)**	**0.161 (0.085-0.262)[a]**	***P*=0.0034**
F/UCr [µg/mmol][3]	39.71 (32.24-47.61)[b]	24.98 (20.94-36.92)[a]	33.11 (15.28-39.33)	*P*=0.0181
E/UCr [µg/mmol][3]	36.36 (29.19-40.08)	27.67 (25.59-44.88)	29.07 (16.66-33.40)	NS
allo-THF/UCr [µg/mmol][3]	1.86 (1.06-2.76)	2.57 (1.54-3.65)	1.48 (0.95-4.24)	NS
THF/UCr [µg/mmol][3]	87.9 (74.4-133.8)	124.6 (61.3-150.4)	113.1 (74.7-185.9)	NS
allo-THE/UCr [µg/mmol][3]	2.92 (2.34-4.75)	2.77 (2.02-5.05)	2.90 (0.96-4.53)	NS
THE/UCr [µg/mmol][3]	296.7 (202.0-378.4)	307.8 (146.0-452.0)	238.8 (147.9-562.1)	NS
**THFs/THEs**	**0.311 (0.251-0.341)[c]**	**0.356 (0.330-0.385)**	**0.418 (0.334-0.473)[a]**	***P*=0.0166**
**allo-THF/F**	**0.0488 (0.0234-0.0726)**	**0.0912 (0.0525-0.1259)**	**0.0985 (0.0442-0.2276)**	**NS**
**THF/F**	**2.30 (1.65-3.38)[c]**	**4.15 (2.75-5.07)**	**5.13 (2.61-7.69)[a]**	***P*=0.0005**
**(THFs + THEs)/UFF**	**88.9 (48.8-124.4)[c]**	**121.7 (72.8-178.1)**	**199.2 (82.0-737.4)[a]**	***P*=0.0291**
**(THFs+THEs+F+E)/UCr**	**470.8 (374.7-578.0)**	**494.3 (258.2-697.7)**	**437.5 (319.1-830.9)**	**NS**
urine volume [ml/24 h]	1540 (1150-1900)	1955 (1360-2720)	2050 (1050-3500)	NS
UCr [mmol/24 h]	9.60 (7.29-10.41)	9.06 (6.23-11.15)	9.01 (7.36-11.08)	NS

Results are presented as medians (interquartile ranges); [1] total plasma steroids, [2] free urinary steroids, [3] total urinary steroids. Free and total urinary steroids are corrected each time for UCr; *comparison was performed with the Kruskal-Wallis test with the post-hoc Dunn's test;

[a] *P*<0.05 compared with a normotensive pregnancy with appropriate for gestational age baby (AGA-NT group = **healthy controls**)

[b] *P*<0.05 compared with a normotensive pregnancy complicated by small for gestational age baby (SGA-NT group)

[c] *P*<0.05 compared with a pre-eclamptic pregnancy complicated by small for gestational age baby (SGA-PE group).

Table S4. Results of the stepwise forward multiple regression conducted for the subpopulation of patients comprising pre-eclamptic women and healthy controls

	PE with AGA baby	PE with SGA baby
**plasma F/E**	R=-0.348; P<0.001	R=-0.398; P<0.001
**UFF/UFE**	R=-0.400; P<0.001	R=-0.393; P<0.001
**THFs/THEs**	NS	R=0.344; P<0.001
**allo-THF/F**	NS	R=0.438; P<0.001
**THF/F**	NS	R=0.538; P<0.001
**(THFs + THEs)/UFF**	R=0.323; P<0.001	R=0.541; P<0.001
**(THFs + THEs + F + E)/UCr**	NS	NS

Results were reported only when the models were significant (P<0.05). In the created models, the calculated parameter (e.g., plasma F/E) was considered as a dependent variable (each parameter was used in a separate model) while the independent variables were: maternal age, pre-pregnancy BMI, GA at sampling, hypothyroidism, gestational diabetes, infant's sex, nulliparity, presence of PE with AGA baby, presence of PE with SGA baby.

Table S5. Results of the stepwise forward multiple regression conducted for the subpopulation of women with FEMALE fetuses comprising women with SGA baby and healthy controls

	SGA baby in normotensive pregnancy	SGA baby in pre-eclamptic pregnancy
**plasma F/E**	NS	R=-0.504; P=0.001
**UFF/UFE**	NS	R=-0.332; P=0.021
**THFs/THEs**	R=0.286; P=0.039	R=0.467; P=0.001
**allo-THF/F**	NS	R=0.694; P<0.001
**THF/F**	NS	R=0.558; P<0.001
**(THFs + THEs)/UFF**	NS	R=0.694; P<0.001
**(THFs + THEs + F + E)/UCr**	NS	NS

Results were reported only when the models were significant (P<0.05). In the created models, the calculated parameter (e.g., plasma F/E) was considered as a dependent variable (each parameter was used in a separate model) while the independent variables were: maternal age, pre-pregnancy BMI, GA at sampling, hypothyroidism, gestational diabetes, infant's sex, nulliparity, fact of giving birth to the SGA baby in normotensive pregnancy, fact of giving birth to the SGA baby in pre-eclamptic pregnancy.

Table S6. Results of the stepwise forward multiple regression conducted for the subpopulation of women with MALE fetuses comprising women with SGA baby and healthy controls

	**SGA baby in normotensive pregnancy**	**SGA baby in pre-eclamptic pregnancy**
**plasma F/E**	*R=-0.247; P=0.073*	R=-0.320; *P*=0.022
**UFF/UFE**	R=-0.297; *P*=0.021	R=-0.379; *P*=0.004
**THFs/THEs**	NS	R=0.302; *P*=0.032
**allo-THF/F**	NS	NS
**THF/F**	*R=0.201; P=0.060*	R=0.506; *P*<0.001
**(THFs + THEs)/UFF**	NS	R=0.468; *P*<0.001
**(THFs + THEs + F + E)/UCr**	NS	NS

Results were reported only when the models were significant (*P*<0.05). In the created models, the calculated parameter (e.g., plasma F/E) was considered as a dependent variable (each parameter was used in a separate model) while the independent variables were: maternal age, pre-pregnancy BMI, GA at sampling, hypothyroidism, gestational diabetes, infant's sex, nulliparity, fact of giving birth to the SGA baby in normotensive pregnancy, fact of giving birth to the SGA baby in pre-eclamptic pregnancy.

Fig S1. Differences in the values of UFF/UFE in the study groups, with respect to the fetal sex. Boxplots present: medians (middle points), interquartile range (box), and range. Outliers were excluded according to Tuckey's method.

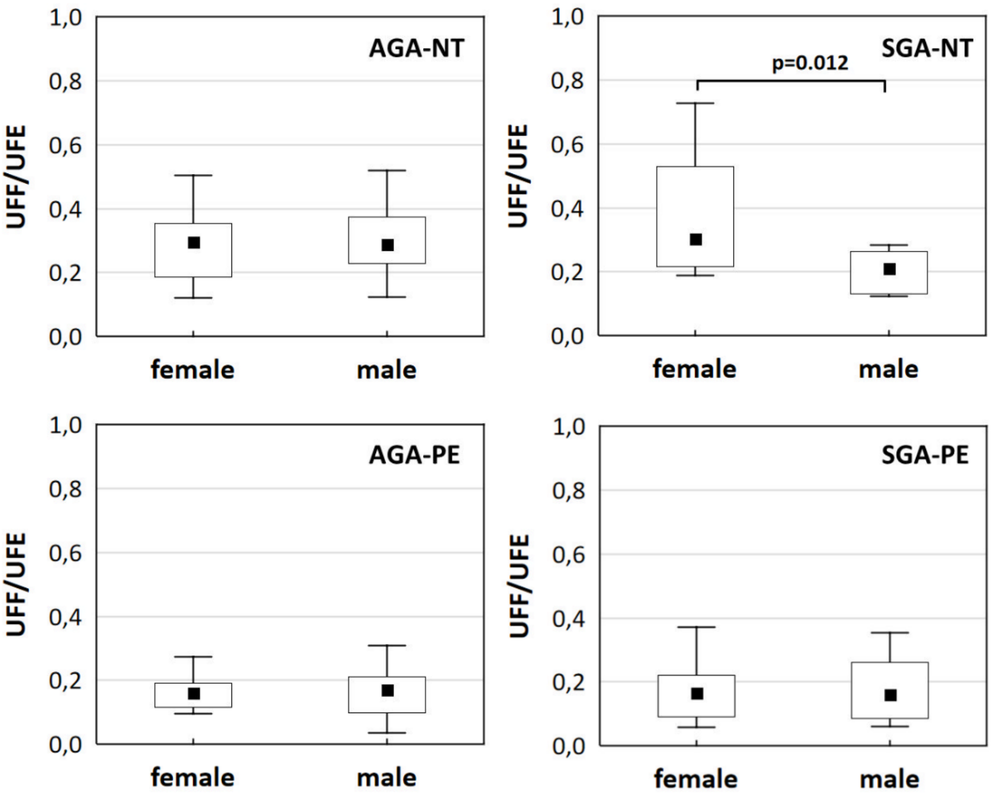

Fig S2. Differences in the values of THFs/THEs in the study groups, with respect to the fetal sex. Boxplots present: medians (middle points), interquartile range (box), and range. Outliers were excluded according to Tuckey's method.

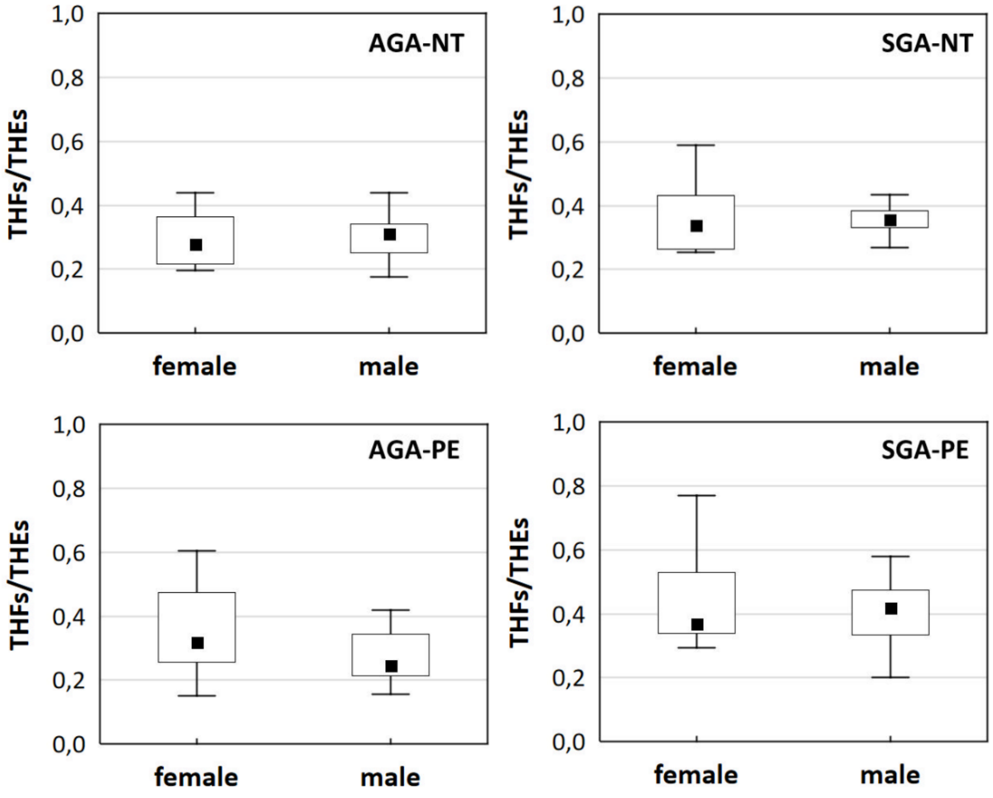

Fig S3. Differences in the values of allo-THF/F in the study groups, with respect to the fetal sex. Boxplots present: medians (middle points), interquartile range (box), and range. Outliers were excluded according to Tuckey's method.

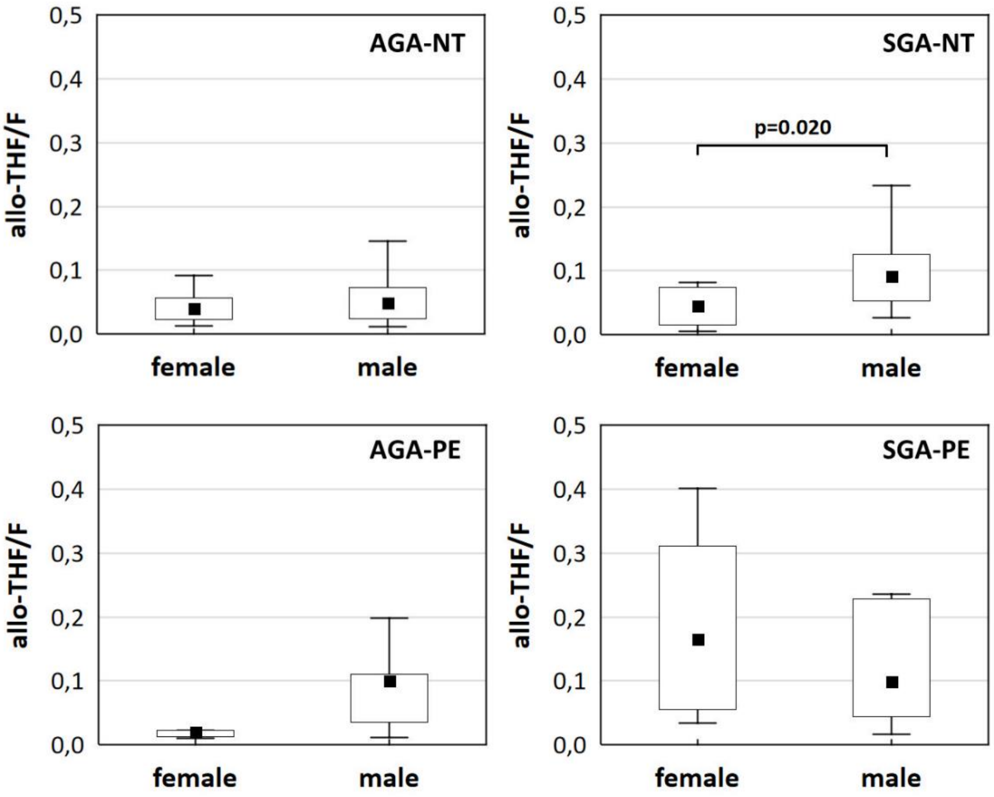

Fig S4. Differences in the values of THF/F in the study groups, with respect to the fetal sex. Boxplots present: medians (middle points), interquartile range (box), and range. Outliers were excluded according to Tuckey's method.

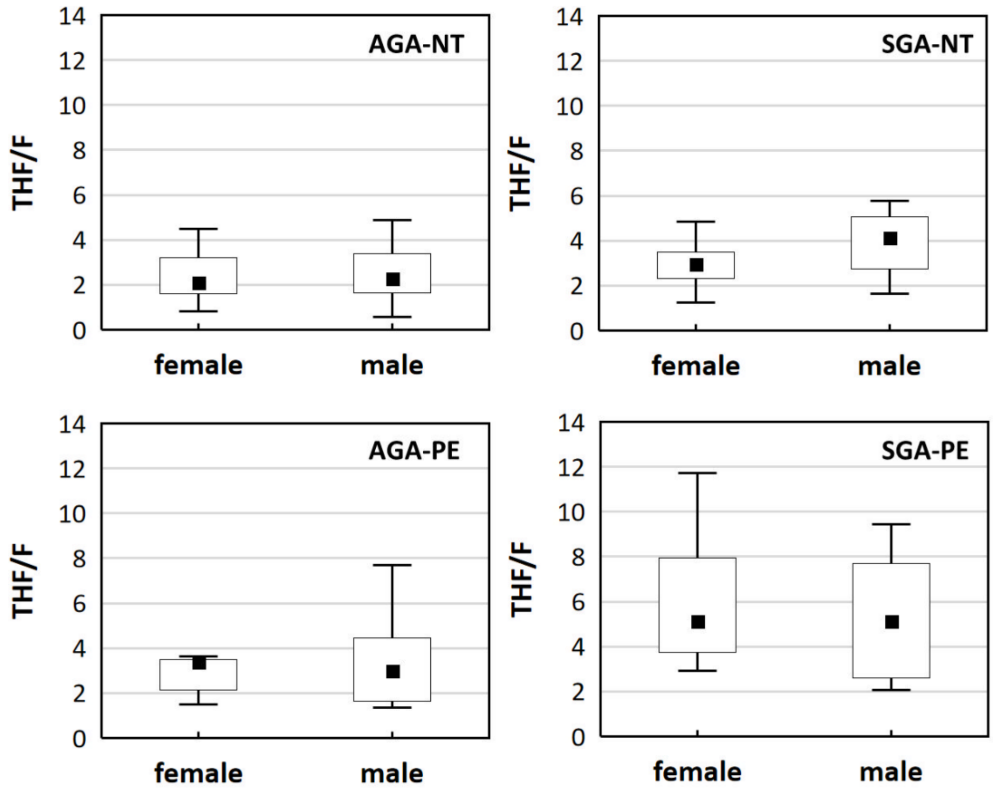

Fig S5. Differences in the values of plasma F/E in the study groups, with respect to the fetal sex. Boxplots present: medians (middle points), interquartile range (box), and range. Outliers were excluded according to Tuckey's method.

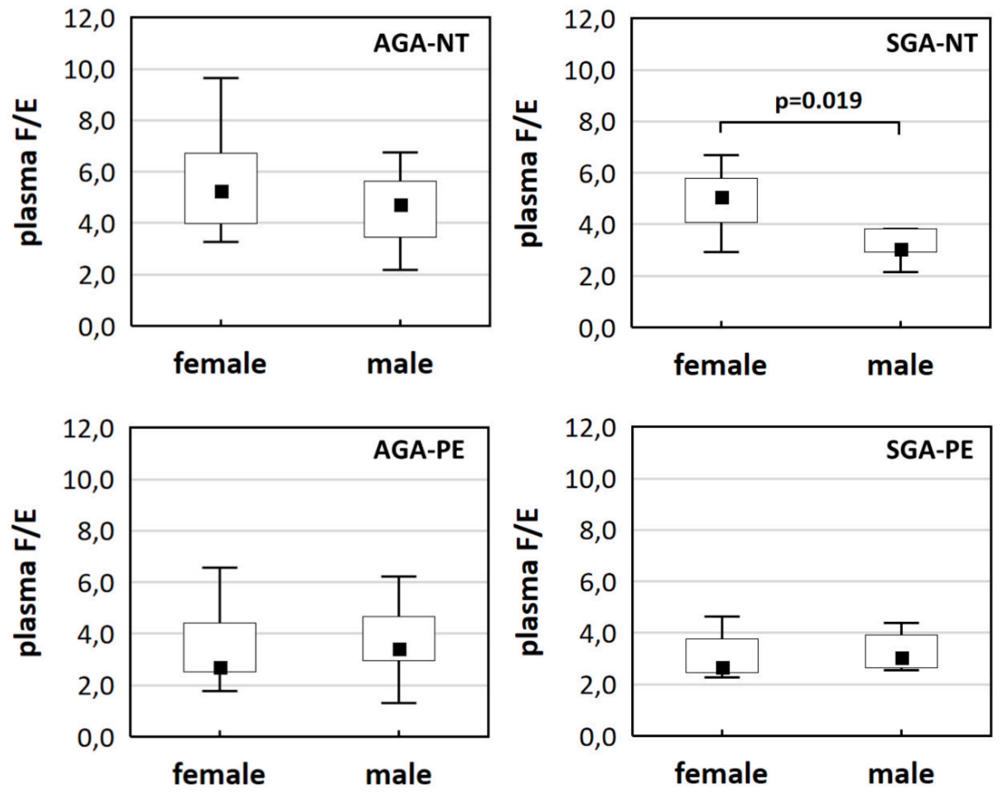

Fig S6. Differences in the values of (THFs+THEs)/UFF (metabolic clearance of F) in the study groups, with respect to the fetal sex. Boxplots present: medians (middle points), interquartile range (box), and range. Outliers were excluded according to Tuckey's method.

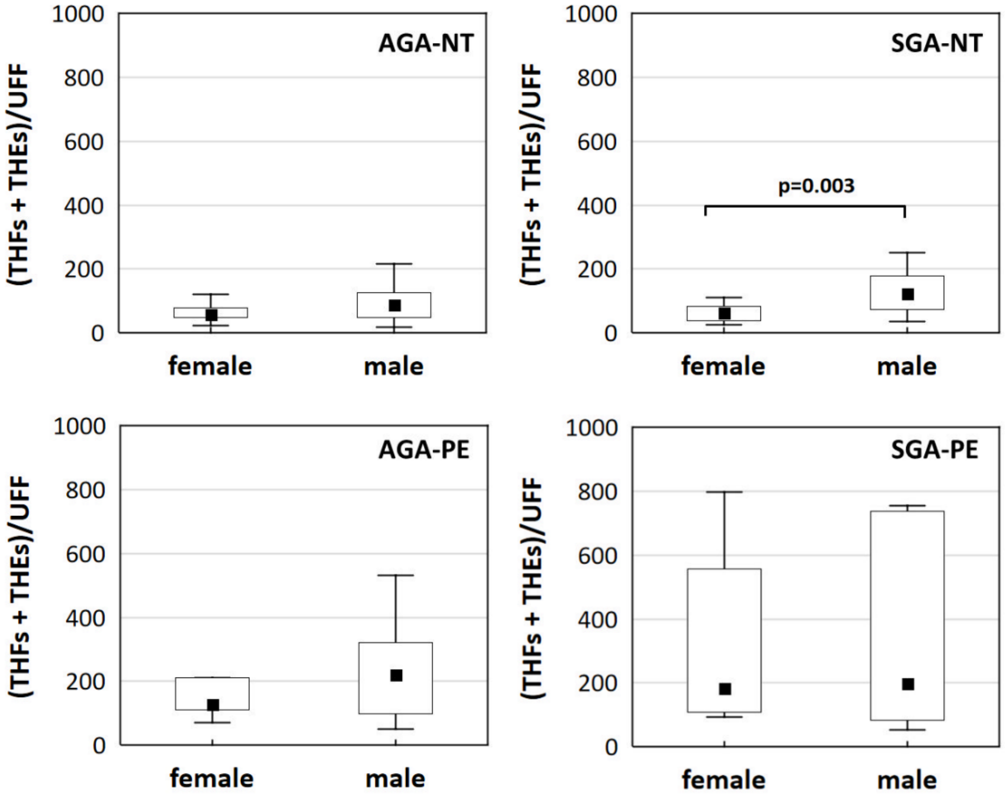

Supplement: Supplementary file 1 — Supplementary Materials [file 41598_2019_54362_MOESM1_ESM.pdf]
